# Correction: An *ab initio* investigation of the structural, mechanical, electronic, optical, and thermoelectric characteristics of novel double perovskite halides Cs_2_CaSnX_6_ (X = Cl, Br, I) for optically influenced RRAM devices

**DOI:** 10.1039/d3ra90038j

**Published:** 2023-04-25

**Authors:** Saira Kiran, Umair Mumtaz, Aymen Mustafa, Muhammad Imran, Fayyaz Hussain, Umbreen Rasheed, R. M. A. Khalil, Ejaz Ahmad Khera, Alia Nazir

**Affiliations:** a Institute of Physics, The Islamia University of Bahawalpur 63100 Pakistan; b Centre of Excellence in Solid State Physics, University of the Punjab Lahore Pakistan; c Department of Physics, Govt. College University Faisalabad 38000 Pakistan imraniub86@gmail.com; d Nishtar Medical University Multan 66000 Pakistan; e Materials Simulation Research Laboratory (MSRL), Institute of Physics, Bahauddin Zakariya University Multan 60800 Pakistan fayyazhussain248@yahoo.com; f Department of Physics Bahawalnagar Campus, The Islamia University of Bahawalpur 63100 Pakistan

## Abstract

Correction for ‘An *ab initio* investigation of the structural, mechanical, electronic, optical, and thermoelectric characteristics of novel double perovskite halides Cs_2_CaSnX_6_ (X = Cl, Br, I) for optically influenced RRAM devices’ by Saira Kiran *et al.*, *RSC Adv.*, 2023, **13**, 11192–11200, https://doi.org/10.1039/D3RA00078H.

The authors regret that the email address for Muhammad Imran: imraniub86@gmail.com, was incorrectly listed under affiliation *d*. The corrected list of affiliations is as shown above.

The Royal Society of Chemistry apologises for these errors and any consequent inconvenience to authors and readers.

## Supplementary Material

